# THE ASSOCIATION BETWEEN PATIENT-LEVEL PROACTIVITY AND RISK OF CANCER-RELATED FALLS

**DOI:** 10.1093/geroni/igae098.3370

**Published:** 2024-12-31

**Authors:** Chuan Lu, Qianyan Zheng

**Affiliations:** Dana Farber Cancer Institute, Boston, Massachusetts, United States; Harvard T. H. Chan School of Public Health, Boston, Massachusetts, United States

## Abstract

Cancer-related falls are prevalent among elder patients given physiological and functional compromise undergoing formidable antineoplastic treatment process. However, effective fall prevention can be challenging without a clear risk-navigating system and sufficient patient self-awareness. The purpose of this cross-sectional study was to evaluate fall-related burden and identify potential risk factors among elders undergoing active cancer treatment, based on the public Medicare Health Outcome Survey (MHOS) collecting patient-reported outcomes provided by Medicare health programs in the United States. The study compared the distributions of clinically implemented fall prevention and patient proactive consultation regarding fall management using beneficiaries from 2018 (14937; 6.55%) to 2022 (21683; 7.74%) undergoing active cancer treatment. Multivariable-adjusted logistic regressions were performed using 2022 cohort to explore potential risk factors of falls. Fall occurrence rates sustained around 30%. Patient’s consultation rate revealed a significant growth over the 5-year period (2018: 29.39% vs. 2022: 34.27%; p< 0.001), while prevention implementation rates were without significant change. Lung cancer (OR=1.995, 95%CI=1.803-2.208) and colon cancer (OR=1.648, 95%CI=1.478-1.837) were associated with greater risk of falls compared to non-cancers. Patients who were advised to increase physical activeness (OR=0.719, 95%CI=0.664-0.779) and with active fall consultants (OR=0.171, 95%CI=0.157-0.186) exhibited significantly lower fall occurrences. This study substantiated the potential role of proactive measures, namely promoting patient’s physical activeness and risk awareness, in reducing fall risk among elders under antineoplastic treatment. Colon or lung cancer may be priority risk identifiers for fall prevention. Future prospective trials are warranted to establish clearer clinical guidance to minimize fall risks targeting elder cancer populations.

